# Pathophysiological Characteristics of Diabetic Ocular Complications in Spontaneously Diabetic Torii Rat

**DOI:** 10.1155/2010/615641

**Published:** 2010-05-25

**Authors:** Tomohiko Sasase

**Affiliations:** Biological/Pharmacological Research Laboratories, Central Pharmaceutical Research Institute, Japan Tobacco Inc., 1-1 Murasaki-cho, Takatsuki, Osaka 569-1125, Japan

## Abstract

The Spontaneously Diabetic Torii (SDT) rat, a nonobese type 2 diabetes model, develops severe diabetic retinopathy as result of chronic severe hyperglycemia. Although existing diabetes animal models also develop ocular complications, severe retinal lesions frequently observed in human diabetes patients such as preretinal neovascularization or retinal detachment are not found. Distinctive features in SDT rat are hypermature cataract, tractional retinal detachment with fibrous proliferation, and massive hemorrhaging in the anterior chamber. These pathophysiological changes are caused by sustained hyperglycemic condition and subsequent increased expression of vascular endothelial growth factor (VEGF) in retina, iris, and ciliary body. Although some differences in diabetic retinopathy exist between SDT rats and humans (e.g., a low incidence of neovascular formation and poor development of nonperfused area are found in this animal), SDT rat will be a useful model in studies of the pathogenesis and treatment of diabetic retinopathy.

## 1. Introduction

Many animal models have been used in research into diabetes mellitus (DM) and its complications. Animal models of chemically induced DM such as streptozotocin (STZ) or alloxan-induced diabetic animals are widely used [1]. Genetic models of DM such as Nonobese diabetic (NOD) mice [2], Bio-Breeding (BB) rats [3], *ob/ob* mice [4], *db/db* mice [5], Goto-Kakizaki (GK) rats [6], Zucker diabetic fatty (ZDF) rats [7], and Otsuka Long-Evans Tokushima fatty (OLETF) rats [8] are also used commonly. Although these model animals develop either type 1 (T1D) or type 2 (T2D) diabetes and subsequent ocular complications, severe retinal lesions frequently observed in human diabetes patients such as preretinal neovascularization or retinal detachment are not found; early pathological changes such as pericyte loss [9, 10], early biophysiological changes such as retinal leukostasis [11], and abnormal pattern in electroretinogram (ERG) [12] are observed, at most. 

The Spontaneously Diabetic Torii (SDT) rat, a nonobese T2D model is an inbred rat strain established from Sprague-Dawley (SD) rat by Shinohara et al. [13, 14]. As a result of chronic severe hyperglycemia, SDT rats develop diabetic retinopathy (DR) [13–20], diabetic peripheral neuropathy [19, 20], and diabetic nephropathy [21]. Of these, severe DR such as cataract, tractional retinal detachment with fibrous proliferation, and massive hemorrhaging in the anterior chamber is a distinctive feature of SDT rat [13, 14]. Since the establishment and the first report of SDT rat by Shinohara et al. [13], dozens of papers have been published. In the present short paper, pathophysiological characteristics of ocular complications in SDT rat are outlined.

## 2. General Characteristics

**Figure 1:**
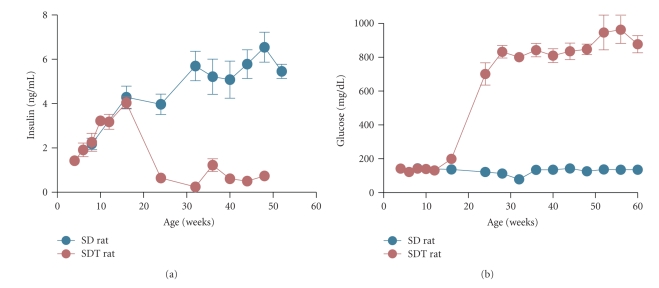
Nonfasting plasma insulin and glucose levels in Spontaneously Diabetic Torii (SDT) rats and control Sprague-Dawley (SD) rats. Diminish of pancreatic *β*-cells evokes hypoinsulinemia (a) and subsequent severe hyperglycemia (b) in SDT rats. Plasma glucose levels sharply increase at 15–20 weeks of age and eventually reach a plateau, approximately 800 mg/dL. Each value represents mean ± S.E.M. (*N *= 6–8).

Male SDT rats exhibit noticeable hyperglycemia, polyuria, and glucosuria concomitant with diminished blood insulin levels and decreased body weight by 15–20 weeks of age (Figure 1). The cumulative incidence of diabetes reaches 100% up to 40 weeks of age. In contrast, the incidence is 33% in female SDT rats [13]. Preceding the onset of diabetes, glucose intolerance with impaired insulin secretion [13, 22] and impaired lipid catabolism [23] are also observed. Genetic analysis for diabetes in SDT rats identified significant quantitative trait loci (QTL) (*Gisdt1*, *Gisdt2*, and *Gisdt3*) for glucose intolerance on rat chromosomes 1, 2, and X, respectively, indicating that the diabetic features in SDT rat are polygenically inherited [24]. Hyperglycemia in SDT rat is spontaneously developed, predominantly due to an insulin secretory defect resulting from pathological damage to the pancreatic islets, especially *β*-cells [22, 25]. Following primary microvascular events in the pancreatic islet such as congestion and hemorrhage (8–10 weeks), inflammation, progressive fibrosis (10–20 weeks), and atrophy with diminished *β*-cells (38 weeks) are observed [22]. These inflammations are different from autoimmune-mediated inflammation observed in autoimmune diabetes. A major locus on chromosome 3 (*Dmsdt1*) was identified as a dominantly acting SDT allele that induces islet inflammation and fibrosis [26].

## 3. Ocular Pathology

### 3.1. Lens

**Figure 2:**
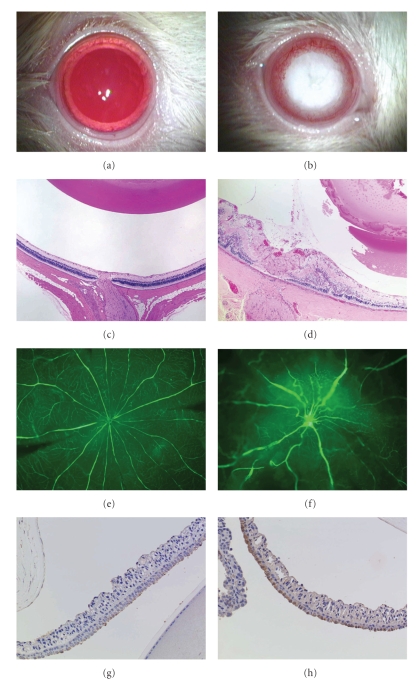
Typical ocular observations in control male SD rats (a, c, e, and g) and SDT rats (b, d, f, and h). (a) and (b) macroscopic photographs of eyes. Hypermature cataract is observed 100% in male SDT rats by 40 weeks of age. (c) and (d) histopathological changes in lens and retina at 80 weeks old. Proliferative fibrovascular membrane in vitreous is found around the optic disk. Retina is locally thickened and formed a fold. Disintegration of lens is also observed. H&E stain. (e) and (f) fluorescein angiomicroscopy at 80 weeks of age. Abnormal vascular formation, including venous dilation and meandering vascular networks, is characteristically observed in SDT rats. Extensive fluorescein leakage is found around the optic disk. (g) and (h) anti-VEGF staining of iris at 80 weeks of age. VEGF immunoreactivity is increased in iris of SDT rats. This may cause a massive hemorrhage on iris in some severe cases.

Cataract is the most frequently occurring ocular complication in DM and is also often observed in diabetic animal models with certain disease duration (e.g., STZ-induced diabetic rats, ZDF rats). In male SDT rats, macroscopic opacity of lens is observed by 40 weeks of age (Figures 2(a) and 2(b)) [13]. Lens clouding begins at posterior pole of lens and finally progresses to mature cataract. Histopathologically, hypermature cortical cataract is suggested by severe swelling, vacuolation, liquefaction, disintegration of the lens fibers, and Morgani's globules in the lens cortex. Nuclear sclerosis and opacified lens cortex are observed, and lens rupture is also found at this stage [18]. Because of this mature cataract, fundus could not be examined by ophthalmoscopy. Cataract in SDT rats was completely prevented by glycemic control such as chronic insulin treatment [17, 19, 20] and pancreatic transplantation [27]. In addition, the histopathological changes of lens were preceded by an increase in lens sorbitol content (our unpublished data). These findings clearly indicate that cataract in SDT rat is due to sustained hyperglycemia.

### 3.2. Vitreous Body

Proliferative diabetic retinopathy (PDR) concerns new vessels extending into the vitreous cavity and causing fibrovascular proliferation, retinal detachment, and vitreous hemorrhage. In SDT rats, the vitreous body was shrunken and cortex was detached from the retina. Proliferative fibrovascular membrane was formed between the folded retina and the distorted lens (Figures 2(c) and 2(d)) [13, 16, 18]. Fibrovascular membrane was infiltrated with inflammatory cells, and capillary vessels found in the fibrovascular membrane had thin walls. Vitreous hemorrhages were observed in some severe cases [18].

### 3.3. Retina

Among the many diabetic animal models, severe retinal abnormality is a prominent feature of SDT rat. In SDT rats, retina was locally thickened and formed retinal folds and was detached from retinal pigmented epithelium. These tractional changes of retina were observed only at the center of retina, never at the peripheral retina (Figures 2(c) and 2(d)) [13, 15, 16, 18]. Immunostaining for albumin showed marked leakage at the site of the tractional retinal detachment, suggesting hyperpermeability of the retinal vessels around the detachment site [16]. Fluorescein retinal flat-mount of SDT rats showed abnormal vascular formation, including venous dilation and meandering vascular networks (Figures 2(e) and 2(f)) [16, 17]. Dilated retinal vessels and a newly formed capillary network were also evident pathologically. Slight hemosiderin deposition was found in the retinal ganglion cell layer [16, 18]. Acellular capillaries and pericyte loss were observed in trypsin digestion preparation; however, capillary microaneurysms were not evident [16, 17]. Vascular nonperfusion area, bleeding, or hard/soft exudates were not observed in flat-mount preparations of retina from SDT rats even at over 80 weeks of age [15].

### 3.4. Iris

Although the neovascular glaucoma is frequently observed in human DR, iris neovascularization has not been reported among diabetic animal models. Iris neovascularization was found in some cases in SDT rats. Fibrovascular tissue covering the pupil or anterior lens capsule may induce pupillary block or angle-closure glaucoma. In a severe case, massive hemorrhage was found in the anterior chamber [13, 16, 18]. Increased aqueous humor vascular endothelial growth factor (VEGF) level [17], which may be derived from anti-VEGF antibody positive ciliary epithelium [28] and iris (Figures 2(g) and 2(h)), presumably causes iris neovascularization and subsequent bleeding from the neovasculature into the anterior chamber.

## 4. Electroretinogram (ERG)

ERG is used to detect abnormal function of the retina. Prolongation of the peak latency in ERG is a very early alteration in diabetic patients, even in patients having no ophthalmoscopically visible alterations at this stage [29, 30]. STZ-induced diabetic rats show prolongation of peak latency in ERG, and the prolongation is prevented with insulin treatment [31, 32]. SDT rats also showed retinal dysfunction in ERG earlier than histopathological changes [17, 33]. Both amplitudes and implicit times of the ERG in SDT rats were not significantly different from those of SD rats at prediabetic stage. However, at postdiabetic 44 weeks of age, amplitudes of the *a*− and *b*−waves and the oscillatory potentials (OPs) were reduced with prolonged implicit times in SDT rats. Because OPs are preferentially decreased in human DR, this is a differing characteristic between DR of humans and SDT rats. The prolonged implicit times and decreased amplitudes of OPs were clearly prevented with long-term treatment of insulin [17], PKC*β*-specific inhibitor, JTT-010 [20], or an angiotensin II receptor blocker (ARB), candesartan [34]. Therefore, depressed ERG in SDT rat is caused by chronic hyperglycemia and may reflect vascular and neuronal damage of retina, as frequently observed in human DR.

## 5. Vascular Endothelial Growth Factor (VEGF) and Pigment Epithelium-Derived Factor (PEDF)

VEGF plays an important role in retinal neovascularization and hyperpermeability [35, 36]. Clinical observations indicate that VEGF concentration in ocular fluid positively correlates with neovascularization activity in DR [37, 38]. In patients with DR and macular edema, increase of vitreous and/or aqueous humor VEGF concentration has been reported [38, 39]. Increased VEGF mRNA expression and anti-VEGF staining area (e.g., retinal vessels, ganglion cell layer, inner plexiform layer, outer plexiform layer, and retinal pigment epithelium cells) in the retina of SDT rat are reported [15, 40]. Increased aqueous humor VEGF level in SDT rats tended to reduce by glycemic control with insulin treatment [17]. Candesartan decreased the expression of VEGF mRNA by reducing the accumulation of advanced glycation end products (AGEs) [34] and NADPH oxidase [41] in SDT rats. Gene transfer of the soluble form of VEGF receptor Flt-1 (s*flt-1*), the endogenous specific inhibitor of VEGF, into retina with adeno-associated viral (rAAV5) vectors prevented the progress of DR in SDT rats [42]. Therefore, the increased expression of VEGF induced by sustained hyperglycemia is thought to be causally related to DR include retinal neovascularization in SDT rat.

On the other hand, expression of pigment epithelium-derived factor (PEDF), a potent inhibitor of ocular angiogenesis [43], is also upregulated in plasma of SDT rat [44], and anti-PEDF antibody positive cells are detected in retinal vessels, nerve fiber layer, ganglion cell layer, inner plexiform layer, inner nuclear cell layer, outer plexiform layer, and retinal pigment epithelium cells in SDT rats [40]. These findings that VEGF and PEDF expressions were both up-regulated in SDT rat retinae are different from the human DR with low levels of PEDF [45], and the high PEDF levels in retina may have contributed to a low incidence of neovascular formation and poor development of nonperfused area in DR of SDT rat.

## 6. Conclusion

Human DR is characterized by microaneurysms, intraretinal punctate hemorrhages, and macular edema. At advanced stage, nonperfusion area and subsequent neovascularization of the retina, which may extend into the vitreous cavity, proliferation of fibrous tissue, vitreous hemorrhage, and retinal detachment, are frequently found. To study and develop treatment for DR, animal models with DR resembling human DR are desperately needed. The earliest histopathological signs of DR such as selective loss of intramural pericytes from retinal capillaries, capillary dilation, and varicose loop formation are frequently observed in some diabetic animal models (e.g., STZ-induced diabetic rats) [46]. Meanwhile, SDT rats develop severe ocular complications such as neovascularization and tractional changes in retina. These findings are the main differences between SDT rat and other rodent diabetes models (Table 1). The tractional changes caused by vitreoretinal interaction are similar to those that occur in human PDR. There is no rodent model with DR of such severity; therefore, SDT rat may be the best candidate for research into DR. One of the possible reasons why retinal neovascularization is observed characteristically in SDT rat is the survival period without glycemic control. In our laboratory, SDT rats survived more than 90 weeks without insulin therapy. Under chronic hyperglycemic circumstances, retina is exposed to high concentration of VEGF over a long period. These factors may cause thickening of the posterior vitreous cortex or modified vitreo-retinal interaction [47] in SDT rats. Meanwhile, in contrast to patients with DR, SDT rats did not develop vascular nonperfusion, bleeding, or hard/soft exudates in retina [15]. These may be crucial differences between humans and rodents. Upregulation of PEDF may be a possible cause of low incidence of neovascular formation and nonperfusion area in SDT rat [40]. 

In addition to retinal pathology, there are some other similarities and differences in ocular pathology between diabetic patients and SDT rats (Table 1). Senile cataract is accelerated in diabetics; however, true diabetic cataract is rare condition, occurring typically in young people with acute diabetes. On the other hand, SDT rats and other diabetic animals such as STZ-induced diabetic rats show typical true diabetic cataract. The difference may be caused by rapid progression of extreme hyperglycemia in these diabetic animal models. 

When the aqueous humor does not drain properly by neovasculatures, increased intraocular pressure results in glaucoma. Iris neovascularization and subsequent development of neovascular glaucoma are serious consequences for patients with PDR. Although diabetes may act as a risk factor of open-angle glaucoma [48], there are no diabetic animal models of spontaneously progress iris neovascularization. However hyperpermeability in iris vessels and decreased iris blood perfusion caused by iris vascular endothelial dysfunction are reported [49, 50], iris neovascularization was not found in STZ-induced diabetic rats. Since SDT rat shows iris neovascularization and bleeding from the neovasculature in some severe cases, SDT rat is a useful model of diabetic rubeosis and is expected as a model of diabetic glaucoma [13, 14]. 

Although DR in SDT rat differs from human DR in some respects, these ocular lesions are much more severe than those observed in other diabetic models and were clearly prevented by glycemic control [17, 19, 20, 27]. To accelerate the development of diabetes and its complications in SDT rats, Masuyama et al. established SDT.Cg-*Lepr^fa^* congenic rats (SDT fatty rats) by introducing an *fa* allele of the leptin receptor gene of Zucker Fatty rat into the genome of SDT rats [51]. Onset of diabetes in SDT fatty rat is accelerated to 5 weeks of age by developing adiposity and insulin resistance. Diabetic complications also develop at younger age than in SDT rat [52]. 

In conclusion, severe ocular complications are distinctive features of SDT rat and were “diabetic”, although there are some differences from human DR. In view of the present situation, that there are no ideal animal models of human DR, SDT rat will be useful animal model in studies of the pathogenesis of DR.

## Figures and Tables

**Table 1:** Ocular pathological findings in diabetic human, SDT rats, and STZ-induced diabetic rats.

	Human	SDT rats	STZ rats
Retina			
Hyperpermeability	Yes	Yes	Yes
Retinal detachment	Yes	Yes	No
Retinal thickness	Yes	Yes	Yes
Avascular area	Yes	No	No
Neovascularization	Yes	Yes	Yes
Retinal microaneurysm	Yes	No	No
Pericyte loss	Yes	Yes	Yes
Vessel abnormality	Yes	Yes	Yes
VEGF expression	High	High	High
PEDF expression	Low	High	High
Vitreous body			
Proliferative membrane	Yes	Yes	No
Iris			
Neovascularization	Yes	Yes	No
Lens			
Cataract	Yes	Yes	Yes
	(senile cataract ≫ true diabetic cataract)	(true diabetic cataract)	(true diabetic cataract)
